# Pig Liver Esterases Hydrolyze Endocannabinoids and Promote Inflammatory Response

**DOI:** 10.3389/fimmu.2021.670427

**Published:** 2021-05-17

**Authors:** Qiongqiong Zhou, Bingfang Yan, Wanying Sun, Qi Chen, Qiling Xiao, Yuncai Xiao, Xiliang Wang, Deshi Shi

**Affiliations:** ^1^ State Key Laboratory of Agricultural Microbiology, College of Veterinary Medicine, Huazhong Agricultural University, Wuhan, China; ^2^ James L. Winkle College of Pharmacy University of Cincinnati, Cincinnati, OH, United States

**Keywords:** Carboxylesterases, pig liver esterase, endocannabinoid, arachidonic acid, prostaglandins, inflammation

## Abstract

Endocannabinoids are endogenous ligands of cannabinoid receptors and activation of these receptors has strong physiological and pathological significance. Structurally, endocannabinoids are esters (e.g., 2-arachidonoylglycerol, 2-AG) or amides (e.g., N-arachidonoylethanolamine, AEA). Hydrolysis of these compounds yields arachidonic acid (AA), a major precursor of proinflammatory mediators such as prostaglandin E_2_. Carboxylesterases are known to hydrolyze esters and amides with high efficiency. CES1, a human **c**arboxyl**es**terase, has been shown to hydrolyze 2-AG, and shares a high sequence identity with pig carboxylesterases: PLE1 and PLE6 (**p**ig **l**iver **e**sterase). The present study was designed to test the hypothesis that PLE1 and PLE6 hydrolyze endocannabinoids and promote inflammatory response. Consistent with the hypothesis, purified PLE1 and PLE6 efficaciously hydrolyzed 2-AG and AEA. PLE6 was 40-fold and 3-fold as active as PLE1 towards 2-AG and AEA, respectively. In addition, both PLE1 and PLE6 were highly sensitive to bis(4-nitrophenyl) phosphate (BNPP), an aryl phosphodiester known to predominately inhibit carboxylesterases. Based on the study with BNPP, PLEs contributed to the hydrolysis of 2-AG by 53.4 to 88.4% among various organs and cells. Critically, exogenous addition or transfection of PLE6 increased the expression and secretion of proinflammatory cytokines in response to the immunostimulant lipopolysaccharide (LPS). This increase was recapitulated in cocultured alveolar macrophages and PLE6 transfected cells in transwells. Finally, BNPP reduced inflammation trigged by LPS accompanied by reduced formation of AA and proinflammatory mediators. These findings define an innovative connection: PLE-endocannabinoid-inflammation. This mechanistic connection signifies critical roles of carboxylesterases in pathophysiological processes related to the metabolism of endocannabinoids.

## Introduction

Carboxylesterases (E.C.3.1.1.1) constitute a class of enzymes critical in drug metabolism, detoxification and lipid mobilization (1–4). These enzymes rapidly hydrolyze carboxylic acid esters, amides and thioesters (2). Carboxylesterases split a drug into two parts and cause large changes in structure, lipophilicity or both (2). As a result, hydrolysis determines the efficacy and toxicity of drugs metabolized by these enzymes. For example, the antiplatelet agent clopidogrel undergoes hydrolysis and its hydrolytic metabolite no longer has antiplatelet activity (5, 6). In contrast, the anti-influenza viral agent oseltamivir requires hydrolysis to exert therapeutic activity (7). Furthermore, hydrolysis of clopidogrel represents detoxification and the opposite is true with oseltamivir (6, 7). In addition to hydrolysis-based detoxification, carboxylesterases interact covalently with organophosphates stoichiometrically (2). Such interactions reduce the amount of organophosphates, which otherwise interact with acetylcholineesterase and cause toxicity.

Carboxylesterases belong to the superfamily of α/β fold hydrolases (8, 9). This superfamily includes structurally similar enzymes such as lipases. For many years, carboxylesterases have been recognized to have lipase activity (10–18). Indeed, several carboxylesterases have been shown to hydrolyze triglycerides and cholesterol esters (11, 12). While hydrolysis of lipids is considered to favor lipid elimination, we and others have shown that overexpression of carboxylesterases leads to lipid accumulation instead (19–22). It has been assumed that the action of carboxylesterases participates in remodeling lipid droplets, favoring lipid retentions (20). In addition, hydrolysis of lipid compounds has been implicated in signal transduction, notably endocannabinoids (23–25): 2-arachidonoylglycerol (2-AG) and N-arachidonoylethanolamine (AEA). Those are two major endogenous ligands of cannabinoid receptors and activation of these receptors exerts a variety of biological activities in a wide range of organs such as the brain, liver, spleen, lungs, and small intestine (26).

AEA and 2-AG are traditionally established to be hydrolyzed by monoacylglycerol lipase (MAGL), fatty acid amide hydrolase and α/β fold hydrolase 6/12 (ABHD6/12) (27, 28). However, several studies have demonstrated a strong involvement of carboxylesterases in their hydrolysis. In human THP1 cells (a monocytic cell line), MAGL and carboxylesterases contribute 32-40% and 40-50% to the hydrolysis of 2-AG (29, 30), respectively. The involvement of carboxylesterases is implicated in mice (30). Hydrolysis of these endocannabinoids leads to the formation of arachidonic acid (AA). This metabolite undergoes oxidation by cyclooxygenase 2 and is converted into prostaglandins (PGs), potent proinflammatory mediators (30–32). These findings suggest that carboxylesterases play critical roles in inflammatory responses.

All mammalian species studied, without exceptions, express multiple carboxylesterases. However, the number of carboxylesterase genes varies greatly from one species to another. In rodents such as mouse, as many as 20 genes are described (1). In contrast, the human genome has only seven including a pseudogene (1). Nevertheless, two human carboxylesterases, CES1 and CES2, are recognized to play major roles in drug metabolism and nutrient processing (1, 2). CES1 is encoded by two highly identical genes (1A1 and 1A2) (1, 33, 34) whereas CES2, on the other hand, is encoded by a single gene with several alternative splicing variants (35). Based on cDNA cloning-sequencing analysis, pigs express a few carboxylesterases (pig liver esterase, PLE) (36). However, the genomic basis for the multiplicity remains to be established. PLE1 and PLE6 share a high sequence identity with CES1, a human carboxylesterase that efficaciously hydrolyzes 2-AG (36, 37).

Pigs are increasingly important animals for modeling human pathophysiological conditions (38). Critically for this study, the pig immune system resembles man for over 80% in contrast to rodents with only about 10% in terms of anatomy and functions (38). For example, pigs and humans but not rodents have tonsils (38). The aim of the present study was to test the hypothesis that PLE1 and PLE6 efficaciously hydrolyze major endocannabinoids with immunostimulating activity. As expected, both PLE1 and PLE6 hydrolyzed 2-AG and AEA with high efficiency. In addition, both carboxylesterases were highly sensitive to bis(4-nitrophenyl) phosphate (BNPP), an established serine enzyme inhibitor predominately toward carboxylesterases (39–41). Based on the inhibitory study with BNPP, PLEs contributed to the hydrolysis of 2-AG by 33.1 to 88.4% among various organs and cells. To ascertain the functional significance of the hydrolysis, exogenous addition or transfection of PLE6 increased the expression and secretion of proinflammatory cytokines when cells were treated with the immunostimulant lipopolysaccharide (LPS). This increase was recapitulated in both *in vitro* and *in vivo* experiments. These findings define an innovative connection: PLE-endocannabinoid-inflammation. This mechanistic connection signifies critical roles of carboxylesterases in important pathophysiological processes related to the metabolism of endocannabinoids.

## Results

### Hydrolysis of Major Endocannabinoids by PLE1 and PLE6

**Figure 1:**
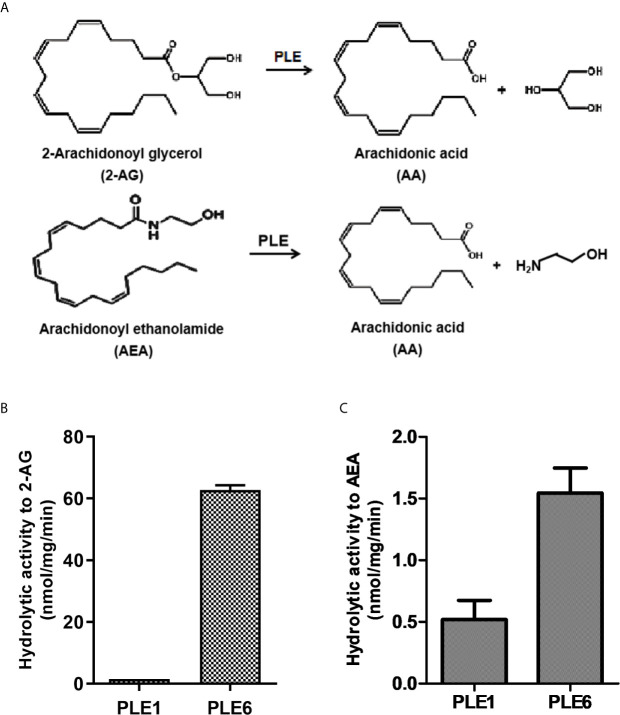
Hydrolysis of 2-AG and AEA by purified PLE1 and PLE6. **(A)** Reaction diagram for 2-AG and AEA hydrolysis. **(B)** Hydrolysis of 2-AG. **(C)** Hydrolysis of AEA. Substrates (2-AG or AEA) at 200 µM was prepared in Tris-HCl buffer (50 mM, pH 7.4) and then mixed with purified PLE1 or PLE6 (10 µg). After incubation for 10 min at 37°C, the reactions were terminated, and the formation of AA was monitored with LC-MS/MS. The data in Figures 1B and C are presented as the mean ± standard error of the mean (SEM) of 3 independent experiments.

PLE1 and PLE6 are abundant pig carboxylesterases and have been shown to hydrolyze foreign compounds such as amoxicillin (42). This study was performed to test whether these enzymes hydrolyze endogenous signaling compounds such as endocannabinoids (**Figure 1A**), essential molecular species in the endocannabinoid system (26–28). To test this possibility, PLE1 and PLE6 were expressed in a heterologous system and purified to homogeneity as described in our previous publications (36, 42). The homogeneity was confirmed by SDS-PAGE (sodium dodecyl sulfate-polyacrylamide gel electrophoresis) followed by Coomassie blue staining (**Figures S1A**, **1B**). Western blotting, with purified PLE1 and PLE6 as the standards, showed that PLEs were highly abundant in the liver S9 fractions (**Figure S1C**).

To gain catalytic insight, purified PLE1 and PLE6 were tested for the hydrolysis of the standard substrate *p*-nitrophenylacetate (*p*-NPA) and two major endocannabinoids: 2-AG and AEA (**Figures S1** and **1**). As shown in **Figures S1D, E**, both PLE1 and PLE6 hydrolyzed *p*-NPA with a comparable specific activity: 1.70 and 2.13 µmol/mg/min, respectively. Liver S9 fractions had a specific rate of 5.98 µmol/mg/min (**Figure S1E**), suggesting that PLE1 and PLE6 together represented the major hydrolytic activity in the liver towards *p*-NPA. Likewise, both PLE1 and PLE6 hydrolyzed 2-AG and AEA (**Figures 1B, C**). However, the relative specific rates varied greatly depending on an enzyme and a substrate. PLE1 had a specific rate of 1.56 nmol/mg/min, while PLE6 had a rate of 62.70 nmol/mg/min (**Figure 1B**), representing a 40-fold difference. Similarly, both PLE1 and PLE6 hydrolyzed AEA. PLE1 had a specific rate of 0.52 nmol/mg/min whereas PLE6 had 1.54 nmol/mg/min (**Figure 1C**). Overall, 2-AG was a better substrate for both enzymes (**Figures 1B, C**).

Next we tested whether PLE1 and PLE6 differ in the sensitivity towards BNPP, an aryl phosphodiester known to inhibit carboxylesterases with high efficacy (39–41). As shown in **Figure S2**, BNPP caused a concentration-dependent inhibition of both PLE1 and PLE6. However, the relative sensitivity varied considerably. PLE6 had an estimated IC_50_ value of 10 µM, whereas PLE1 had 0.1 µM (**Figure S2A**), representing a 100-fold difference in the relative sensitivity. However, comparable inhibitions of both enzymes were detected with higher concentrations of BNPP. For example, BNPP at 100 µM inhibited PLE6 by 66.0% and PLE1 by 75.4%, respectively (**Figure S2A**). As expected, liver S9 fractions hydrolyzed both 2-AG (2.94 nmol/mg/min) and AEA (0.20 nmol/mg/min). The hydrolysis of 2-AG and AEA by liver S9 fractions was inhibited significantly by BNPP (**Figure S2B**) with 2-AG being inhibited by 53.4% whereas AEA by 75.0%. These results suggest that PLE1 and PLE6 are major hepatic enzymes in hydrolyzing these endocannabinoids. Finally, BNPP (0.1-100 µM) caused little cytotoxicity (**Figure S2C**).

### Inverse Regulation of Inflammation by 2-AG and PLE

AEA and 2-AG, 2-AG in particular, are two major molecular species in the cannabinoid signaling and exert a wide range of pathophysiological functions including inflammatory responses (23–26). We next tested potential roles of PLEs in the inflammation. The focus was on PLE6 and 2-AG based on the high relative activity of this enzyme toward this cannabinoid (**Figure 1B**). Two types of primary cells were used: pig alveolar macrophages (PAMs) and pig hepatocytes (PHCs). PAMs have strong immune significance, whereas PHCs have strong metabolizing functions, although both types of cells share these functions (43–46). As shown in **Figures S3A, B**, primary cells with high purity were isolated and cultured. Western blotting detected abundant expression of PLEs in both PAMs and PHCs with the latter expressing to a much greater extent (**Figure S3C**). Critically, homogenates of PAMs and PHCs effectively hydrolyzed 2-AG at a specific rate of 4.47 nmol/min/mg and 3.25 nmol/min/mg, respectively. BNPP (100 µM) profoundly inhibited 2-AG hydrolysis in both PAM (77.3%) and PHC (88.4%) homogenates (**Figures S3D, E**).

**Figure 2:**
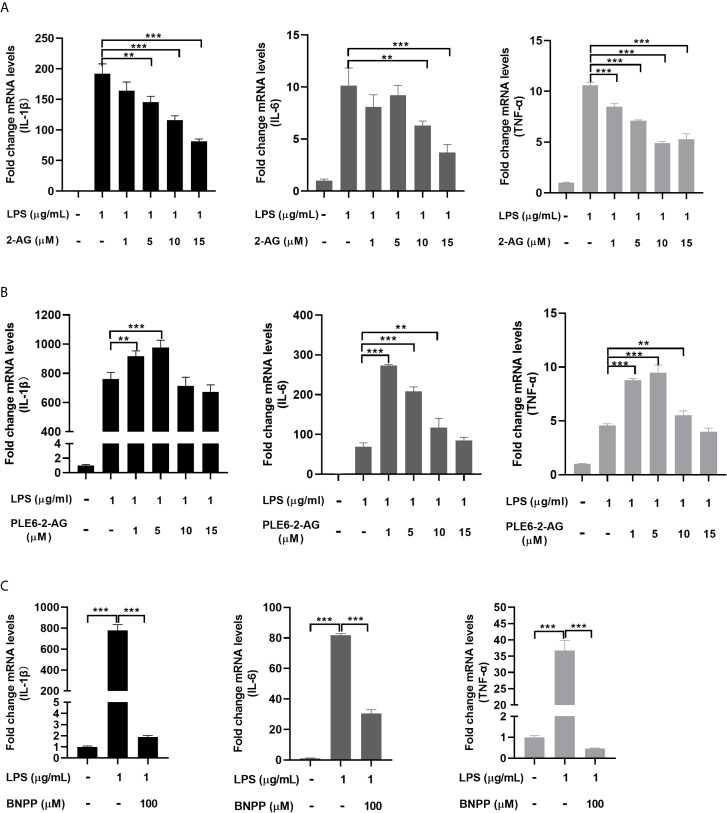
Effect of 2-AG, PLE6-hydrolyzed 2-AG and BNPP on the LPS-stimulated mRNA expression of proinflammatory cytokines in PAMs. **(A)** Effect of 2-AG on the LPS-stimulated mRNA expression of proinflammatory cytokines. PAMs were cultured and incubated with LPS (1 µg/mL) and 2-AG at various concentration (1 µM, 5 µM, 10 µM, 15 µM) for 24 h. Total RNA was isolated and analyzed for the mRNA level of IL-1β, IL-6 and TNF-α by RT-qPCR. **(B)** Effect of PLE6 hydrolyzed 2-AG on the LPS-stimulated mRNA expression of proinflammatory cytokines. PAMs were cultured and incubated with LPS (1 µg/mL) and a 0.5-h preincubation mixture of PLE6 with 2-AG at various concentration (1 µM, 5 µM, 10 µM, 15 µM) for 24 h. Total RNA was isolated and analyzed for the mRNA level of IL-1β, IL-6 and TNF-α by RT-qPCR. **(C)** Effect of BNPP on the LPS-stimulated mRNA expression of proinflammatory cytokines. PAMs were cultured and preincubated with BNPP (100 µM) for 3 h, and then LPS (1 µg/mL) was treated for another 24 h. The levels of inflammatory factors in the cell lysates were detected. Statistical significance was indicated by asterisks (**P < 0.01; ***P < 0.001).

Next we tested whether the immunostimulant LPS triggers immune response in PAMs and whether the action of PLE6 promotes the response. As expected, LPS greatly upregulated the mRNA expression of multiple proinflammatory cytokines such as IL-1β (interleukin-1β), IL-6 (interleukin-6) and TNF-α (tumor necrosis factor-α) (**Figure 2**). Importantly, the upregulation was attenuated by 2-AG in a concentration-dependent manner (**Figure 2A**). To specify whether hydrolysis is involved in the attenuation, 2-AG was pre-incubated with PLE6 for 30 min and the pre-incubation mixtures were tested for the attenuation activity. As shown in **Figure 2B**, the attenuation was significantly decreased with the pre-incubation mixture (**Figure 2B**). Actually, the pre-incubation mixtures with lower concentrations of 2-AG (e.g., 1 µM) increased the expression of proinflammatory cytokines (**Figure 2B**). On the other hand, the pre-incubation mixtures with increased concentrations of 2-AG (e.g., 10 µM for IL-1β) was less effective (**Figure 2B**). It was likely that the amount of PLE6 and the pre-incubation time used here were not sufficient to completely hydrolyze the amount of 2-AG at higher concentrations. Consistent with this notion, BNPP (100 µM) significantly suppressed LPS-induced expression of proinflammatory cytokines in PAMs (**Figure 2C**). These results conclude that 2-AG inhibits inflammatory responses in PAMs and hydrolysis of 2-AG by PLE6 reverses the inhibition.

### Enhanced Inflammatory Response by PLE1 or PLE6 With Endogenous 2-AG

**Figure 3:**
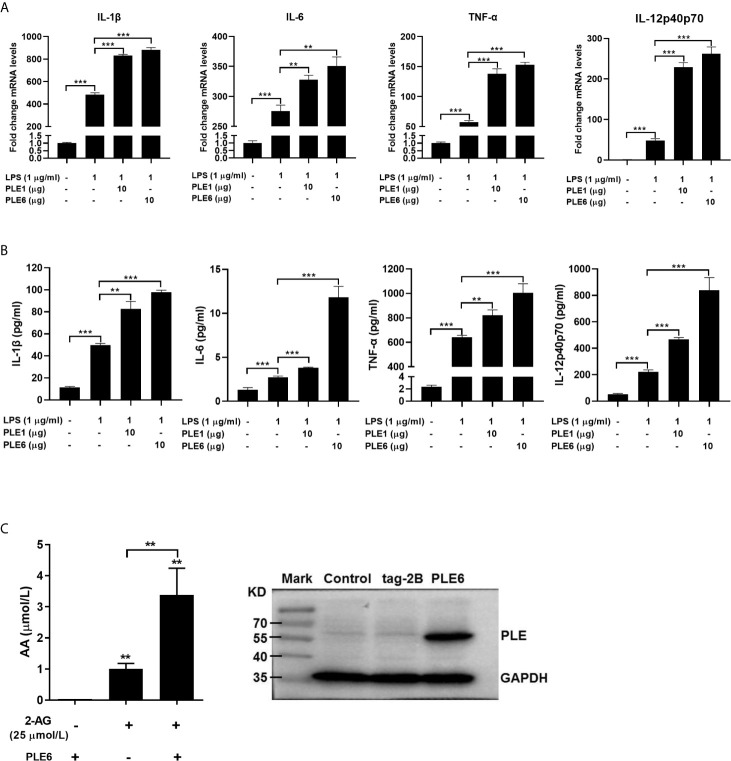
Enhanced LPS-stimulation on the expression of proinflammatory cytokines in PAMs by addition of PLE1/6 or increased AA secretion in transfected PLE6. **(A)** Enhanced LPS-stimulation on the mRNA expression of proinflammatory cytokines in PAMs by addition of purified PLE1 or PLE6. PAMs were cultured for 24 h, and then treated with LPS (1 µg/ml) and PLE1 or PLE6 (10 µg). After incubation for additional 6 h, total RNA was isolated and analyzed for the mRNA level of proinflammatory cytokines by RT-qPCR. **(B)** Enhanced LPS-stimulation on the protein expression of proinflammatory cytokines in PAMs. Cells were treated as Experiment A (above). However, culture supernatant was collected and analyzed by protein chip. **(C)** Increased AA secretion by PLE6 transfected cells. 293T cells were transfected with pCMV-tag-2B-PLE6 or the corresponding vector. The transfected cells were treated with 2-AG (25 µM). After treatment for 1 h, the supernatant was extracted and analyzed for the level of AA by LC-MS/MS (Left). The expression of transfected PLE6 was confirmed by Western blotting (Right). Data are presented as the mean ± SEM of 3 independent experiments. Statistical significance was indicated by asterisks (** P < 0.01; *** P < 0.001).

LPS has been shown by many investigators to induce release of 2-AG (31, 47, 48). Next we tested whether cultured PAMs incubated with purified PLE1 or PLE6 without addition of 2-AG increases the expression of proinflammatory cytokines stimulated by LPS. As shown in **Figures 3A, B**, both RT-qPCR and protein chip demonstrated that PLE1 and PLE6 further enhanced the levels of IL-1β, IL-6, TNF-α and IL-12 in PAMs. These results suggest that LPS induced the release of 2-AG some of which was secreted into media and hydrolyzed by PLEs, eventually leading to increased expression of inflammatory cytokines.

To complement the experiment with added exogenous PLEs (extracellular), we next tested whether overexpression of PLE6 through transfection increases the hydrolysis of 2-AG. Cells (293T) were transfected to overexpress PLE6, treated with 2-AG and monitored for its hydrolysis. The selection of 293T cells was based on two important considerations: (a) 293T cells support high-levels of transfection efficiency and (b) these cells express little carboxylesterases (49). As shown in **Figure 3C** (Left), transfection of PLE6 increased the hydrolysis of 2-AG (increased formation of AA) by as many as 3-fold. Transfection indeed resulted in increased expression of PLE6 (Right of **Figure 3C**). These results conclude that exogenous and endogenous PLE6 both increases 2-AG hydrolysis, signifying enhanced inflammatory response.

### Enhanced Proinflammatory Response by PLEs in Coculture Models

**Figure 4:**
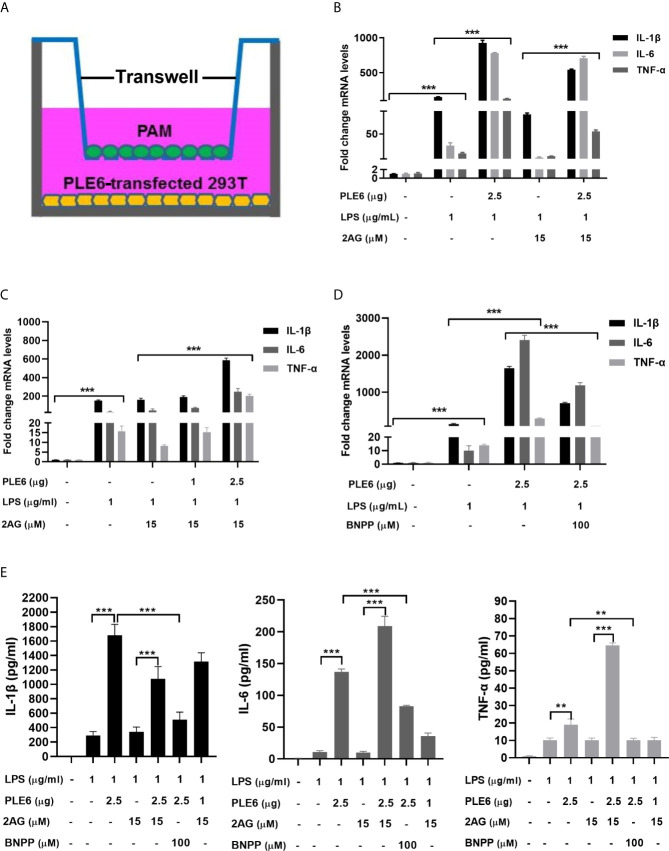
Enhanced LPS-stimulation on the expression of proinflammatory cytokines in coculture model of PAMs and 293T cells. **(A)** Diagrammatic presentation of the coculture model. **(B)** Enhanced LPS-stimulation on the expression of proinflammatory cytokines in coculture model. PAM and PLE6-transfected 293T cells (non-transfected cells as controls) were cocultured for 24 h along with LPS (1 µg/mL), 2-AG (15 µM) or both. total RNA was isolated and analyzed for the mRNA level of proinflammatory cytokines by RT-qPCR. **(C)** Enhanced LPS-stimulation on the expression of proinflammatory cytokines in coculture model as a function of the amount of PLE6. Coculture was performed as described for Experiment B (above). However, the transfection of PLE6 plasmid was performed at 1 and 2.5 µg. **(D)** Reduced PLE6 increases on the expression of proinflammatory cytokines by BNPP. Coculture was performed similarly as described. However, cells were cocultured in the presence of BNPP at 100 µM. Likewise, the mRNA expression of proinflammatory cytokines was determined. **(E)** Effect of PLE6, 2-AG, BNPP or in combination on protein expression of proinflammatory cytokines in the coculture model. Cells were cultured and treated similarly. The levels of proinflammatory cytokines was determined with a protein chip. The data in **Figure 4** are presented as the mean ± SEM of 3 independent experiments. Statistical significance was indicated by asterisks (** P < 0.01; *** P < 0.001).

The increases of LPS-induced proinflammatory cytokines were further studied with double-layered cocultures. This was of significance as it specifies whether the hydrolytic metabolites by PLEs travels from one compartment to another, signifying distance effect *in vivo*. As structured in **Figure 4A**, PAM cells were cultured in the insert whereas the PLE6 transfected cells were seeded at the bottom of the external compartment. As anticipated, transfection of PLE6 enhanced LPS-induced expression of proinflammatory cytokines, and reduced the 2-AG-decreased expression of proinflammatory cytokines (**Figures 4B, C**). The reductions were also confirmed by BNPP, an PLE6 inhibitor (**Figures 4D**, **S2A**). It should be noted that similar patterns of changes were detected on the protein levels of the pro-inflammatory cytokines (**Figure 4E**).

**Figure 5:**
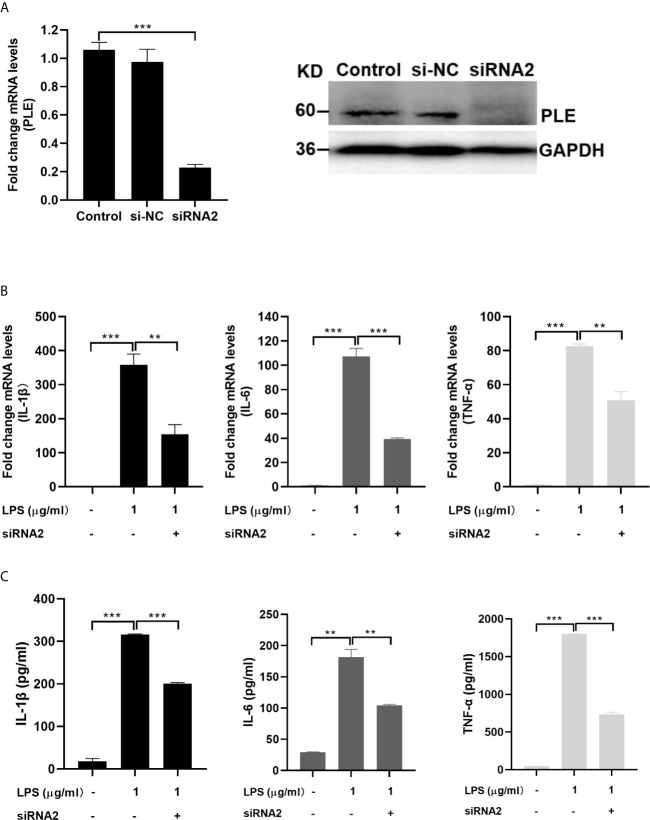
Decreased LPS-stimulation on the expression of proinflammatory cytokines in PAMs by transfection of siRNA2 targeting PLEs. **(A)** The expression of PLEs was confirmed by RT-qPCR (left) and western blotting (Right). si-NC: si negative control. **(B)** Decreased LPS-stimulation on the mRNA expression of proinflammatory cytokines in PAMs by transfection of siRNA2 targeting PLEs. PAMs were cultured for 24 h, and then transfected with siRNA. After incubation for 24 h, LPS (1 µg/mL) was treated for additional 6 **h** Then, total RNA was isolated and analyzed for the mRNA level of proinflammatory cytokines by RT-qPCR. **(C)** Decreased LPS-stimulation on the protein expression of proinflammatory cytokines in PAMs by transfection of siRNA2 targeting PLEs. Cells were treated as Experiment B (above). However, culture supernatant was collected and analyzed by ELISA. Data are presented as the mean ± SEM of 3 independent experiments. Statistical significance was indicated by asterisks (** P < 0.01; *** P < 0.001).

In addition, the role of endogenous PLEs in LPS-induced proinflammatory cytokines was assessed in PAMs transfected with siRNA negative control (siNC) or siRNAs targeting the PLEs. Notably, the results on the levels of mRNA and the corresponding proteins of the proinflammatory cytokines induced by LPS were attenuated by siRNA and the PLEs levels were down-regulated by siRNA, respectively (**Figure 5**), indicating that siRNA are effective in the cells. Consistently, cotransfection of PLE1 or PLE6 with the corresponding siRNA significantly abolished the effect of PLEs overexpression on the levels of proinflammatory cytokines, pointing to the specificity of this experiment (**Figure S4**).

### Association of PLEs With Tissue Injury and Inflammation *In Vivo*

To gain insight into *in vivo* consequences of PLEs-enhanced inflammation, pigs (1 month old, male) were treated with LPS to induce inflammatory responses with or without BNPP, and cell injury and levels of inflammatory cytokines as well as the overall whole body response were monitored. Indeed, LPS-treated pigs initially (1-6 h) exhibited labored breathing, lethargy and reduced feed intake. Subsequently, pigs became lateral decubitus, hypersalivation, shivering and severe dyspnea. These symptoms gradually disappeared after 9 h. The pigs pretreated with BNPP, nevertheless, exhibited lesser severity, notably during the first 4 h right after LPS-treatment, when the symptoms were most severe in pigs treated with LPS alone. It should be noted that the selection of male pigs at an age of 1 month was based on several considerations. Nevertheless, our previous study has shown that there is minimal difference in terms of sex in carboxylesterases (36), and the cost-effective consideration is another factor for the study design with male animals only.

**Figure 6:**
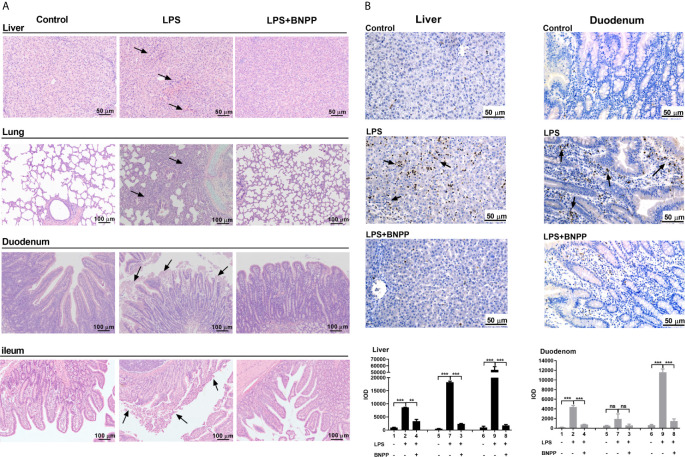
PLE inactivation attenuates tissue injury and the inflammatory response *in vivo*. **(A)** Pathological examination (H&E staining) of pig tissues. Pigs (n=3) were treated with sterile saline or LPS (25 µg/kg) or the combination of LPS and BNPP (25 mg/kg, i.p., given prior to 1 h of LPS induction). Then, tissue injuries were detected after 24 h. **(B)** Neutrophil infiltration was assessed by MPO staining (brown staining), and quantitative analysis was performed by CaseViewer software. Representative images are shown, and the black arrow shows the damage in the tissues. Integral optical density (IOD) is the value of the light absorbed or transmitted by the result of dye staining in different tissue cells under the same illumination condition. The numbers in the figures represent the corresponding pigs. Statistical significance was indicated by asterisks (**P < 0.01; ***P < 0.001).

To gain pathological insight into the observed overall changes induced by LPS alone or LPS-BNPP-cotreatment, pathological examinations were performed. As shown in **Figure 6**, LPS caused necrosis in the liver, disintegration of hepatocytes, thickening of alveolar septa, and shedding and necrosis of intestinal villi were also observed. In contrast, pretreatment with BNPP, an efficacious inhibitor of PLEs provided substantial protection against LPS-induced tissue injury (**Figure 6A**). Neutrophil infiltration in the liver and duodenum, a cellular marker for inflammation, was examined by myeloperoxidase (MPO) staining and integral optical density (IOD). Pretreatment with BNPP offered effective counteractive activity against LPS-induced neutrophil infiltration (**Figure 6B**).

### Decreased Formation of AA and PGs *In Vivo* by Inhibition of PLEs

**Figure 7:**
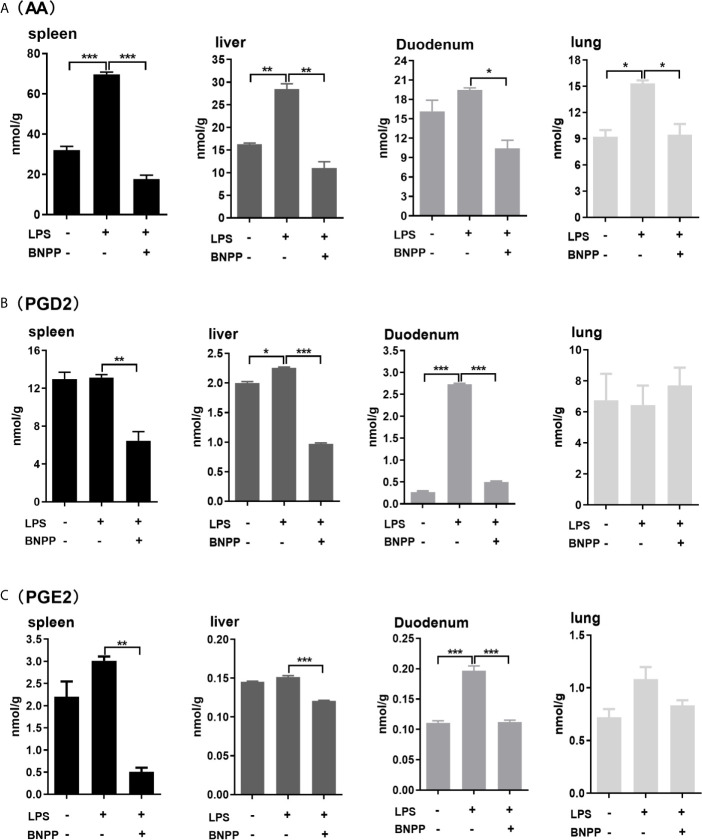
Levels of AA **(A)**, PGD2 **(B)** and PGE2 **(C)** in tissues from pigs treated with BNPP and LPS. Pigs (n = 3) were treated with BNPP (25 mg/kg, i.p.) initially and 1 h later with LPS (25 µg/kg, i.p.) in pigs. Animals were euthanized 24 h after LPS administration. Tissues were harvested, extracted and analyzed for the level of AA, PGD2 and PGE2 with LC-MS/MS. The data in Figure 7 are presented as the mean ± SEM of 3 independent experiments. Statistical significnce was considered at values of P < 0.05 and indicated by an asterisk (*P < 0.05; **P < 0.01; ***P < 0.001).

Hydrolysis of endocannabinoids produces AA, promoting inflammatory response by participating in the differentiation and proliferation of Th1 and Th17 cells (50). Therefore, we next tested whether inhibition of PLEs *in vivo* reduces the formation of AA and its inflammatory effectors: prostaglandin D2 (PGD2) and prostaglandin E2 (PGE2) (30, 32). The results are summarized in **Figure 7**. Among the examined tissues, the spleen, an immune organ, had the highest levels of AA (31.99 nmol/g), PGD2 (12.98 nmol/g) and PGE2 (2.20 nmol/g). Treatment with LPS robustly increased the level of these molecular species. The increases, however, were blunted by BNPP (25 mg/kg) in almost all the examined tissues. These data confirm that PLEs contribute to the AA precursor pool for PGs *in vivo*. It should be noted that replacement of PLE6 transfected cells with primary hepatocytes produced similar results (**Figure S5**), suggesting that the observed distant hydrolysis by carboxylesterases is applicable to *in vivo* situation.

## Discussion

Carboxylesterases have been established to play critical roles in drug metabolism and detoxifications (1, 2). Emerging evidence, nonetheless, has linked the action of these hydrolases to lipid metabolism/processing (3, 4, 10–18). Many lipid species and their precursors are signaling molecules, pointing to regulatory involvement of these hydrolases. In this study, we have shown that PLE6 hydrolyzed with high specific activity the endocannabinoid 2-AG (**Figure 1B**), a major molecular species implicated in an array of physiological functions including behaviors, energy balance, pain and inflammation (23). In addition, PLE6 was sensitive to the predominant carboxylesterase inhibitor BNPP with an IC_50_ value of 10 µM (**Figure S2A**). Critically, addition (extracellular) or transfection (intracellular) of PLE6 increased the expression/secretion of proinflammatory cytokines (**Figures 2**, **3**), and the increase was recapitulated in cocultured alveolar macrophages and PLE6 transfected cells in transwells (**Figure 4**). Finally, inactivation of PLEs by BNPP or knockdown of PLEs by siRNA reduced the magnitude of inflammatory responses trigged by the immunostimulant LPS accompanied by decreased formation of AA, PGD2 and PGE2 (**Figures 5** and **7**). These findings define a new mechanistic connection related to the metabolism of endocannabinoids: PLE-endocannabinoid-inflammation.

**Figure 8:**
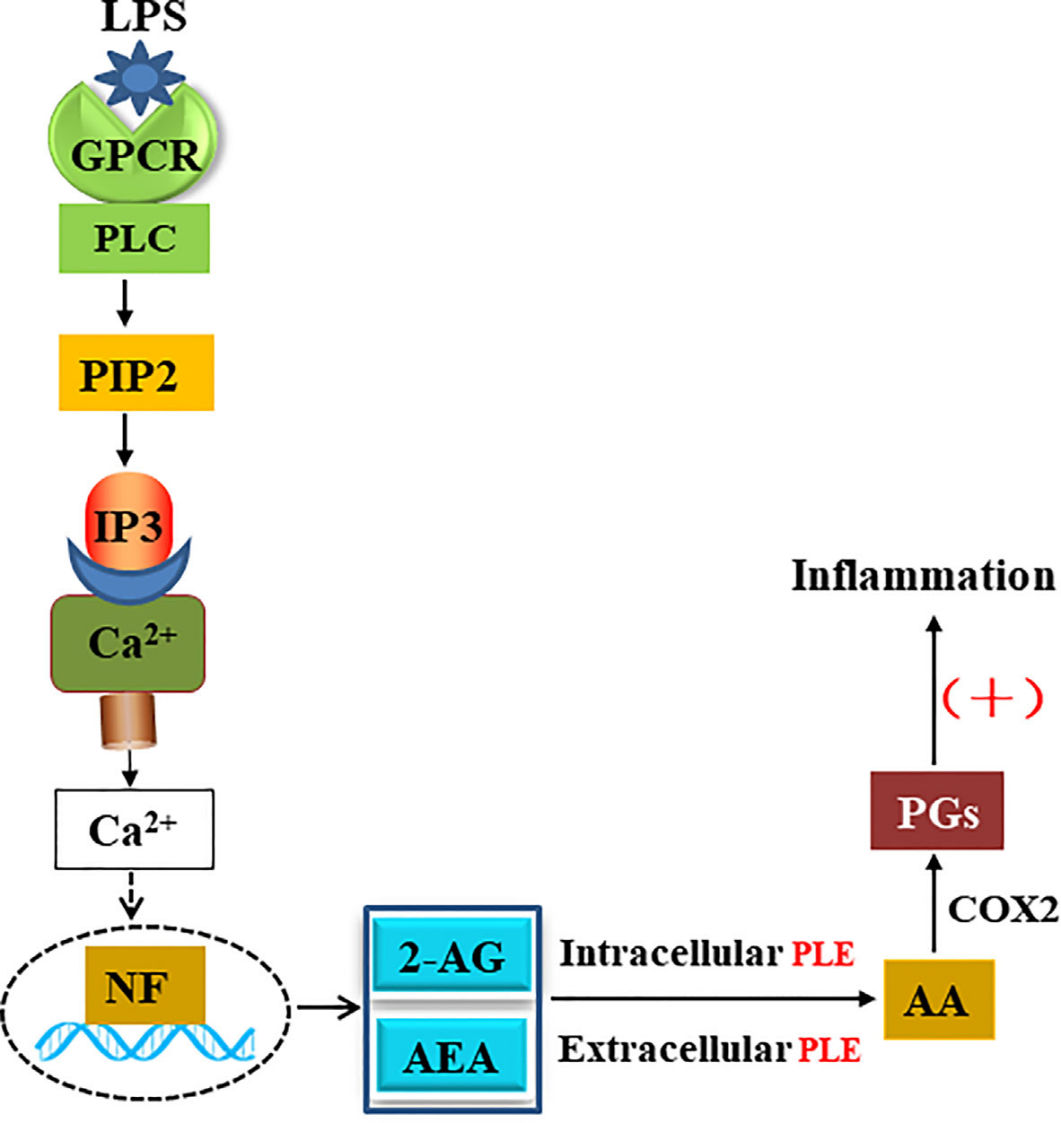
Function of PLEs in eCBs metabolism and inflammatory response.

The carboxylesterases-based mechanistic connection likely operates in species and/or disease dependent manners. It is established that external inflammatory stimuli (e.g., LPS) activates G-protein-coupled receptors (GPCRs) and produces endocannabinoids such as 2-AG through an enzymatic cascade (**Figure 8**). This cascade uses membrane-bound lipids as the resource for endocannabinoids (51). In this study, we have shown that both transfection (intracellular) and addition (extracellular) of PLE6 increased the expression and secretion of proinflammatory cytokines (**Figures 2** **–** **4**). The increase *via* exogenous addition used extracellular 2-AG, presumably serum 2-AG under *in vivo* situation. Indeed, it has been reported that elevated serum 2-AG reflects certain disease conditions such as hypertensive individuals with depression and LPS has been shown by many investigators to induce release of 2-AG (31, 47, 48, 52). Such disease conditions are associated with inflammation, and at least partially through carboxylesterases-based hydrolysis of endocannabinoids.

On the other hand, the abundance of serum carboxylesterases varies depending on a species. Both mouse and rat have abundant presence of carboxylesterases in the blood (1, 53). In contrast, humans have little serum carboxylesterases unless organ injury takes place (54). As described in our previous publication (49), carboxylesterases with an endoplasmic retention signal (HXEL) stay in the endoplasmic reticulum (ER), whereas those without it are secreted into the serum (1, 53). We have reported that pigs had low levels of carboxylesterases detected by a highly cross-recognizing antibody (specific towards multiple carboxylesterases) (36). Given the fact that both PLE1 and PLE6 have the ER-retention signal (**Table S1**), it is likely that the detected carboxylesterases in the serum are neither of the enzymes, or otherwise, the samples were collected under disease conditions.

It is interesting to notice that PLE1 and PLE6, with a sequence identity of 96% at amino acid (**Table S1**), exhibited profound differences in hydrolyzing one substrate but not the other. In this study, we have shown that PLE1 and PLE6 hydrolyzed *p*-NPA at a comparable rate of specific activity: 1.70 versus 2.13 µmol/mg/min, respectively (**Figure S1**). However, they exhibited large differences towards 2-AG and AEA (**Figure 1**). PLE1 hydrolyzed 2-AG at a specific rate of 1.56 nmol/mg/min, while PLE6 at a rate of 62.70 nmol/mg/min (**Figure 1B**), representing a 40-fold difference. Likewise, PLE1 hydrolyzed AEA at a specific rate of 0.52 nmol/mg/min, whereas PLE6 at 1.54 nmol/mg/min (**Figure 1C**), a 3-fold difference. The precise mechanism remains to be determined. Sequence alignment identifies only a total of 18 amino acid substitutions between PLE1 and PLE6 (Figure S6), and half of these substitutions are identical between PLE6 and CES1 (human). CES1 (human) has been shown to effectively hydrolyze endocannabinoids (25, 30). The amino acids shared by PLE6 and CES1 differ markedly from those in PLE1 in terms of charge, hydrophilicity and aromatic side-chain (**Figure S6**). It is assumed that those amino acids shared with CES1 but differed from PLE1 likely contribute significantly to recognizing lipophilic substrates such as endocannabinoids (2-AG) but less so towards relatively hydrophilic substrates such as *p*-NPA.

The lipophilicity-based rationale for the substrate specificity, on the other hand, is not quite applicable to the inhibitor BNPP. Actually, BNPP and *p*-NPA have a similar LogP value (1.7 versus 1.5), an indicator for lipophilicity. However, PLE1 and PLE6 had a 100-fold difference, based on their IC_50_ values, toward BNPP (**Figure S2A**). BNPP is an irreversible carboxylesterase inhibitor (55). It was likely that the contact initiated with PLE1 induced conformational changes favoring the covalent complex in compare with the contact with PLE6. Nevertheless, both pig carboxylesterases, even with PLE6 at an IC_50_ value of 10 µM, were sensitive to BNPP compared with those (e.g., MAGL) known to hydrolyze endocannabinoids (e.g., 2-AG) (55, 56). It was therefore convincible that the concentrations and dose used *in vitro* and *in vivo* in this study had overwhelmingly inhibited PLE6-based hydrolysis (**Figures 2** **–** **7**). Particularly, BNPP reduced the formation of AA and its derivatives by as much as 33.1% to 56.3% among various organs (**Figure 7**) consistent with the relative potency on the inhibited hydrolysis by S9 and culture primary cells (**Figures S2** and **3**). The finding on the formation of AA was largely confirmed on the formation of PGD2 and PGE2 (**Figures 7B, C**). It should be noted that carboxylesterases, compared with other endocannabinoid hydrolases (e.g., MAGL) are abundantly expressed in peripheral organs, particularly the intestine, kidney, liver and lung. As a result, the innovative connection specified in this study, PLE-endocannabinoid-inflammation, signifies reduced inflammatory responses in these organs by inhibiting carboxylesterases. Consistently, previous studies demonstrated that MAGL inhibited cancer growth through hydrolyzing endocannabinoid (57).

In summary, our work points to several important conclusions. First, carboxylesterases are efficacious hydrolases towards endocannabinoids such as 2-AG, constituting alternative mechanisms for the degradation of these biologically active molecules. Second, PLE1 and PLE6, although sharing a 96% sequence identity at amino acid level, differ profoundly in the hydrolysis of endocannabinoids (e.g., 2-AG) (**Table S1**). The parent endocannabinoids and their metabolites have distinct and probably opposite biological functions, notably related to inflammatory response. The slight differences in the sequences of carboxylesterases (e.g., polymorphisms) may have profound clinical implications. Third, we have shown that transfection of PLE6 (intracellular) and addition of purified PLE6 (extracellular) both enhance the expression and secretion of proinflammatory cytokines. This is of significance as serum presence of carboxylesterases varies depending a species and disease condition. And finally, we have shown that BNPP, an inhibitor with high potency towards carboxylesterases, minimizes cell injury *in vivo* induced by the classic immunostimulant LPS. The cannabinoid system exerts a variety of biological activities in a wide range of organs. The results from this study concludes that hydrolysis of these ligands by carboxylesterases has profound implications in terms of their engagement in inflammatory responses.

## Experimental Procedures

### Materials

Provided in the section of **Supplementary Materials** in “Supporting Information”.

### Hydrolysis of *p*-NPA

Described in the section of **Supplementary Materials** in “Supporting Information”.

### Hydrolysis of 2-AG and AEA

Hydrolysis of 2-AG and AEA was performed at 37°C in Tris-HCl (50 mM, pH 7.4) buffer containing substrate concentrations of 200 μM in a total volume of 100 μL. Hydrolysis was initiated by adding purified enzyme (10 μg PLE1 or PLE6), tissue S9 fractions (100 μg) or cell lysates of PAMs/PHCs (20 μg). After 15 min, 30 min or 45 min, reactions were quenched with 200 μL acetonitrile (ACN) containing arachidonic acid-*d_8_* (AA-*d_8_*) (100 ng/mL). Quenched reactions were placed in ice bath for 10 min and then centrifuged at 12,000 rpm (4°C, 10 min). The supernatants (200 μL) were diluted with ACN (800 μL). The hydrolytic metabolite AA was monitored by LC-MS/MS. Control samples were enzymatic reactions stopped at 0 min.

The contribution of PLEs to the hydrolysis of 2-AG and AEA in tissue S9 fractions or in cell lysates was assessed with BNPP (100 μM). Tissue S9 fractions (100 μg) or cell lysates (20 μg) were added to Tris-HCl (50 mM, pH 7.4) buffer along with BNPP (100 μM) in a total volume of 100 μL. After a 10 min-preincubation at 37°C, the reactions were initiated by the addition of 2-AG or AEA (200 μM). After 15 or 30 min, reactions. were quenched with ACN (200 μL) containing AA-*d_8_* (100 ng/mL), placed in ice for 10 min, and centrifuged at 12,000 rpm (4°C, 10 min). Supernatants (200 μL) were transferred, diluted with ACN (800 μL) and analyzed by LC-MS/MS.

### LC-MS/MS Analysis

LC-MS/MS assays were performed using a Waters Xevo G2 QTOF mass spectrometer and Waters Acquity Ultra Performance Liquid Chromatography system (Waters, USA). Samples (1 μL) were injected onto a reverse-phase C18 column (2.1 mm×100 mm, 1.7 μm, Acquity UPLC BEH), and the analytes were eluted with the gradient mobile phase (A: 0.1% formic acid, B: acetonitrile): 0-0.5 min (A-99%, B-1%); 6.5-7.5 min (A-1%, B-99%); 10 min (A-99%, B-1%) with additional 10 min running-time before next injection. The ESI source was set to negative ion mode. The quantifications were based on the standard curves for AA, PGD2 and PGE2 (Cayman). The autosampler was set at 10°C, and the column temperature was at 45°C. The capillary voltage was set to 1 kV, the sampling cone was 30 v and the source temperature was 120°C. N_2_ was used as the desolvation gas with 800 L/h at 450°C, and argon was used as the collision gas with 50 L/h. The collision energy was set to 6.0 eV, and the scanning range was m/z=190-600. Data analysis was performed with Mass Lynx Software Version 4.1. The MS spectrograms of analyte ions were detected as follows: 2-AG, [M + COOH]- m/z=423.275; AEA, [M + COOH]- m/z=392.280; AA, [M - H]- m/z=303.232; AA-*d_8_*, [M - H]- m/z=311.283; PGD2, [M - H]- m/z=351.217; PGE2, [M - H]- m/z=351.217. The polarity of PGE2 was larger and the signal peak appeared earlier than PGD2. The hydrolytic activities of PLEs were quantified with the amount of product AA.

### Transient Transfection

All expression constructs encoding PLE were prepared by inserting cDNA into the pCMV-tag-2B vector. The primers used for the cloning were shown in **Table 1**. Cells (293T) were cultured in Dulbecco’s modified Eagle’s medium containing 1% penicillin-streptomycin and 1% fetal bovine serum. Transfection was performed with Lipofectamine 2000 Reagent (Invitrogen). The expression of transfected PLEs was detected by western blot. To specify the metabolism of 2-AG, 24 h after transfection, 2-AG (25 μM) was added to the medium and incubated for another 1 h. The culture medium was spiked with AA-*d_8_* (100 ng/ml) and extracted with 3 volumes of ethyl acetate containing 0.1% acetic acid. The mixture was vortexed and centrifuged (4°C, 12000 rpm, 10 min). The ethyl acetate layer was recovered and dried under N_2_. The residues were dissolved in 200 μL of acetonitrile and transferred to LC vials for the analysis by LC-MS/MS (liquid chromatography with tandem mass spectrometry).

**Table 1:** The primers for subcloning cDNA of PLE.

Primers	Sequences 5’-3’
pCMV-tag-2B-PLE (F)	GAATTCATGTGGCTTCTCCCGCTGGTCCTGACCTCCCTCG
pCMV-tag-2B-PLE (R)	CTCGAGTCACAGCTCAGCATGCTTTATCTTGGGTGGCTTCTTTGCT

PAMs were cultured in RPIM medium containing 10% fetal bovine serum. siRNA was transfected into PAM with X-treme GENE HP DNA Transfection Reagent (Roche) and the expression of PLEs was detected by western blot after 24h. To specify the effect on the expression of proinflammatory cytokines, the transfected cells (24 h after transfection) were treated with LPS (1 μg/ml) for another 6 h. The culture medium and total RNA were collected and analyzed for the expression (both mRNA and protein) of PLEs and proinflammatory cytokines.

### Quantitative Real-Time PCR (qPCR)

Total RNA was isolated from cells with TRIzol reagent (Invitrogen) and reverse-transcribed (RT) with Superscript Reverse Transcriptase (TaKaRa). RT-qPCR used iQ™ SYBR Green PCR Supermix (TaKaRa) and detected with the Bio-Rad CXF real-time PCR system (Bio-Rad, USA). Primer sequences are shown in **Table 2**. These quences of siRNAs for PLEs are shown in **Table 3**.

**Table 2:** The primer sequences for RT-qPCR.

Genes	Primer sequences (5’-3’)	Fragment size (bps)	Accession number
PLE	F:GGGGATGTGGTGTTTGGTR:TGGGTTTCTTGTCCGATG	121	X63323
GAPDH	F:GAAGGTCGGAGTGAACGGATR:CATGGGTAGAATCATACTGGAACA	149	AF017079
IL-1β	F:GCTGGAGGATATAGACCCCR:GTTGGGGTACAGGGCAGAC	115	*
IL-6	F:ACAAAGCCACCACCCCTAACR:CGTGGACGGCATCAATCTCA	185	*
TNF-α	F:TTCCAGCTGGCCCCTTGAGCR:GAGGGCATTGGCATACCCAC	146	NM-214022

**Table 3:** The sequences of siRNAs for PLEs.

siRNA	Sequences (5’-3’)
siRNA1siRNA2siRNA3si negative control	CCACCUCCUACCCUCCCAUTTAUGGGAGGGUAGGAGGUGGTTCCACCACCUCGGCUGUCUUTTAAGACAGCCGAGGUGGUGGTTGCCGAUGUACGACCAGGAATTUUCCUGGUCGUACAUCGGCTTUUCUCCGAACGUGUCACGUTTACGUGACACGUUCGGAGAATT

### Western Blotting Analysis

Equal amounts of proteins were separated by 12.5% sodium dodecyl sulfate-polyacrylamide gel electrophoresis and transferred electrophoretically to polyvinylidene fluoride membranes. The primary antibody against PLEs, recognizing the common motif, was described in our previous publication (42). The antibody against glyceraldehyde-3-phosphate dehydrogenase was purchased from Proteintech. The secondary Ab was goat anti-rabbit IgG conjugated to horseradish peroxidase, and the proteins recognized by the primary antibody were visualized by the ECL chemiluminescence system (Bio-Rad). The relative intensities were quantified by ImageJ software.

### Isolation and Culture of PHCs and Alveolar Macrophages PAMs

PHCs were isolated from 25-day-old Large White pigs (6 kg, male) according to a modified protocol of Puviani et al. (58). PAMs were collected by bronchoalveolar lavage with phosphate-buffered saline (PBS, pH 7.4) and frozen in liquid nitrogen as the modified protocol previously described (59). Briefly, 30-day-old piglets were euthanized, and lungs were collected. The lungs were washed through a suitable funnel with PBS (pH 7.4) containing penicillin (100 units/mL) and streptomycin (100 µg/mL). Three washings were collected. Cells were centrifuged at 1000 rpm for 10 min and resuspended in RPMI containing 10% (v/v) fetal bovine serum (FBS, Gibco) and 1% penicillin-streptomycin (Gibco). Then, PHCs and PAMs were cultured at 37°C in a humidified incubator with 5% CO_2_. PHCs treated with LPS or 2-AG were cultured with DMEM/high glucose containing 1% FBS, while PAMs treated with LPS or 2-AG were cultured with RPMI containing 1% FBS.

### Preparation of Double-Layered Coculture System

Transwell inserts (polycarbonate membrane, 0.4 µm) were used to construct the double-layered coculture system. PLE6-transfected 293T cells were seeded into 12-well plates at a density of approximately 1×10^6^ cells/well, and PAMs were seeded into transwell polyester membrane inserts at a density of 5×10^5^ cells/well Confluent monolayer cells were formed after a 24 h-culture,. The double-layered coculture system was then prepared in 12-well plates by placing inserts with a confluent PAM layer over the confluent 293T cell layer. The volume of the applied culture medium was 0.5 mL in the apical compartment and 1 ml in the basolateral compartment. After coculture for 24 h, the medium was placed with RPMI containing 1% FBS. BNPP (100 µM) was added to the double-layered coculture system and preincubated for 3 h. Then, LPS (1 µg/mL) or 2-AG (5 µM, 10 µM, 15 µM) was added to the double-layered coculture system and further incubated for 24 h at 37°C in a humidified incubator with 5% CO_2_. PAM and PLE6-transfected 293T cells were harvested for detection of proinflammatory cytokines by RT-qPCR, and medium supernatants were harvested and detected by protein chip.

### *In Vivo* Experiment

Animal experiments were performed in accordance with the Guide for the Care and Use of Laboratory Animals Monitoring Committee of Hubei Province, China, and the protocol was approved by the Committee on the Ethics of Animal Experiments at the College of Veterinary Medicine, Huazhong Agricultural University.

Crossbred healthy pigs (Landrace × Large White, 1 month old, male, 5~7 kg) were purchased from Wuhan Zhaohui Xingda Animal Husbandry Co., Ltd., Hubei Province, China. All pigs were maintained at an ambient temperature of 20-25°C in an environmentally controlled room by air conditioning and illumination (12 h light and dark cycles). Each cage was equipped with a feeder and water nipple to allow free access to food and drinking water. Nine pigs were divided into 3 groups (N=3/group) with each group having the similar average weight. The pigs were fed in their home cages 10 days before beginning of the experiment. BNPP was administered at 25 mg/kg (0.5 ml/kg) by intraperitoneal (i.p.) injection (1 h before LPS induction). An equal volume of saline or proinflammatory agent LPS (25 μg/kg, 0.5 ml/kg, BW) was administered by i.p. injection. Pigs were euthanized 24 h after LPS injection, and organs were collected and frozen in liquid nitrogen.

### Histological and Immunohistochemical Analyses in LPS-Treated Pigs

Histological analysis of tissue damage was assessed by standard hematoxylin and eosin (H&E) staining of tissue sections (5 μm thickness). For immunohistochemical staining of neutrophils in the liver and duodenum, a primary antibody against pig myeloperoxidase was used (Abcam, ab9535).

### Extraction of AA, PGD2 and PGE2

Tissues were weighed and homogenized (45 Hz, 4°C, 10 min) in a mixture of PBS (50 mM, pH 7.4, 2 ml) and hexane:ethyl acetate (2 ml, 1:1 v/v). Hexane:ethyl acetate (1:1 v/v, 6 mL) containing the internal standards (AA-*d_8_*, 100 ng/ml) was added. The mixtures were vortexed and then centrifuged (4°C, 8000 rpm for 10 min). The organic layer was removed, evaporated under a stream of nitrogen and resolubilized in 200 μL of chloroform. Metabolites were quantified by LC-MS/MS.

### Statistical Analysis

Statistical analyses were performed using Microsoft Excel and GraphPad Prism 8 (GraphPad Software, Inc., San Diego, CA, USA). All assays were performed in triplicate, and the data are expressed as the mean ± SEM (standard error of the mean). Statistical significance was evaluated using the two-tailed Student’s t-test, and statistically significant differences are indicated by asterisks as follows: *P < 0.05, **P < 0.01, ***P < 0.001.

## Data Availability Statement

The raw data supporting the conclusions of this article will be made available by the authors, without undue reservation.

## Ethics Statement

The animal study was reviewed and approved by Committee on the Ethics of Animal Experiments at the College of Veterinary Medicine, Huazhong Agricultural University.

## Author Contributions

DS, QZ, and BY designed and coordinated the experiments. WS, QC, QX, YX, and XW performed experiments and analyzed results. DS, QZ, and BY wrote the manuscript. All authors contributed to the article and approved the submitted version.

## Conflict of Interest

The authors declare that the research was conducted in the absence of any commercial or financial relationships that could be construed as a potential conflict of interest.

## Abbreviations

AA, arachidonic acid; ACN, acetonitrile; AEA, N-arachidonoylethanolamine; 2-AG, 2-arachidonoylglycerol; BNPP, Bis(4-nitrophenyl) phosphate; FBS, fetal bovine serum; IL-1β, interleukin-1β; IL-6, interleukin-6; IOD, integral optical density; LC-MS/MS, liquid chromatography with tandem mass spectrometry; MAGL, monoacylglycerol lipase; PAMs, pig alveolar macrophages; PBS, phosphate-buffered saline; PGD2, prostaglandin D2; PGE2, prostaglandin E2; PHCs, pig hepatocytes; PLE, pig liver esterase; LPS, lipopolysaccharide; MPO, myeloperoxidase; *p*-NPA, *p*-nitrophenylacetate; RT-qPCR, reverse transcription-quantitative polymerase chain reaction; TNF-α, tumor necrosis factor-α.
