# Supplementary material for: Pig Liver Esterases Hydrolyze Endocannabinoids and Promote Inflammatory Response
Source: Front Immunol. 2021 May 17;12:670427. doi: 10.3389/fimmu.2021.670427 (PMC8165269; doi:10.3389/fimmu.2021.670427)
Supplement: Supplementary file 1 [file DataSheet_1.pdf]

Supporting Information

Supplemental Materials and Methods

Materials

2-AG, AEA, AA and AA-d_8 were purchased from Cayman Chemicals. *E. coli* LPS (O55:B5), BNPP and *p*-NPA were purchased from Sigma Chemicals (USA). LPS was dissolved in a vehicle of DMEM/high-glucose medium or 0.9% sodium chloride solution, BNPP was dissolved in 0.9% sodium chloride solution, and *p*-NPA was dissolved in ddH_2O. All solutions and reagents used for tissue extraction and LC-MS/MS analysis were of MS grade and purchased from Thermo-Fisher Scientific.

Enzymatic Assays for *para*-nitrophenylacetate (*p*-NPA)

Recombinant PLE1 and PLE6, with the highest expression abundance in Large White pigs and Tongcheng pigs, respectively(1), were expressed with molecular chaperones pGro7 in *E. coli* Origami (DE3) and purified with a His-tag as described in a previous publication(2). Pig liver S9 fractions were harvested and stored in our laboratory(1). The protein concentrations were determined by a BCA Protein Assay Kit (Pierce) according to the manufacturer's protocol. Hydrolytic activities of purified PLEs and liver S9 fractions were spectrophotometrically determined as described in a previous paper(3). Substrate *p*-NPA (200 μM) was prepared in 990 μl of reaction buffer Tris-HCl (50 mM, pH 7.4) and then mixed with purified PLE1, PLE6 (10 μg) or liver S9 fractions (100 μg). Then, the rate of formation of *parp*-nitrophenol (*p*-NP) was monitored continuously at 410 nm by spectrometers.

The inhibition of BNPP for PLEs was also performed with *p*-NPA. Purified PLEs

(10 μg), tissue S9 fractions (100 μg) or cell homogenates of PAMs/PHCs (20 μg) were prepared in Tris-HCl buffer (50 mM, pH 7.4) along with BNPP (100 μM) in a total volume of 100 μL. After preincubation for 10 min at 37 °C, the reactions were initiated by the addition of *p*-NPA (200 μM). Then, the rate of formation of *p*-NP was monitored continuously at 410 nm by spectrometers.

Cytotoxicity Assay

Cells (293T) were cultured in 96-well plates at a density of 10,000 cells/well. After incubation for 12 h, the medium was replaced with reduced serum medium (1%) containing BNPP at various concentrations (0.1-1000 μM), and the treatment lasted for 36 h. CCK-8 reagent of 10 μL (Dojindo, Japan) was then added to each well. The optical density was determined at 450 nm after 2 h incubation at 37 °C, and the final optical density values were expressed by subtracting the background reading (no seeded cells). The cell viability was expressed as the percentage of cells incubated without BNPP.

Supplemental Figure 1

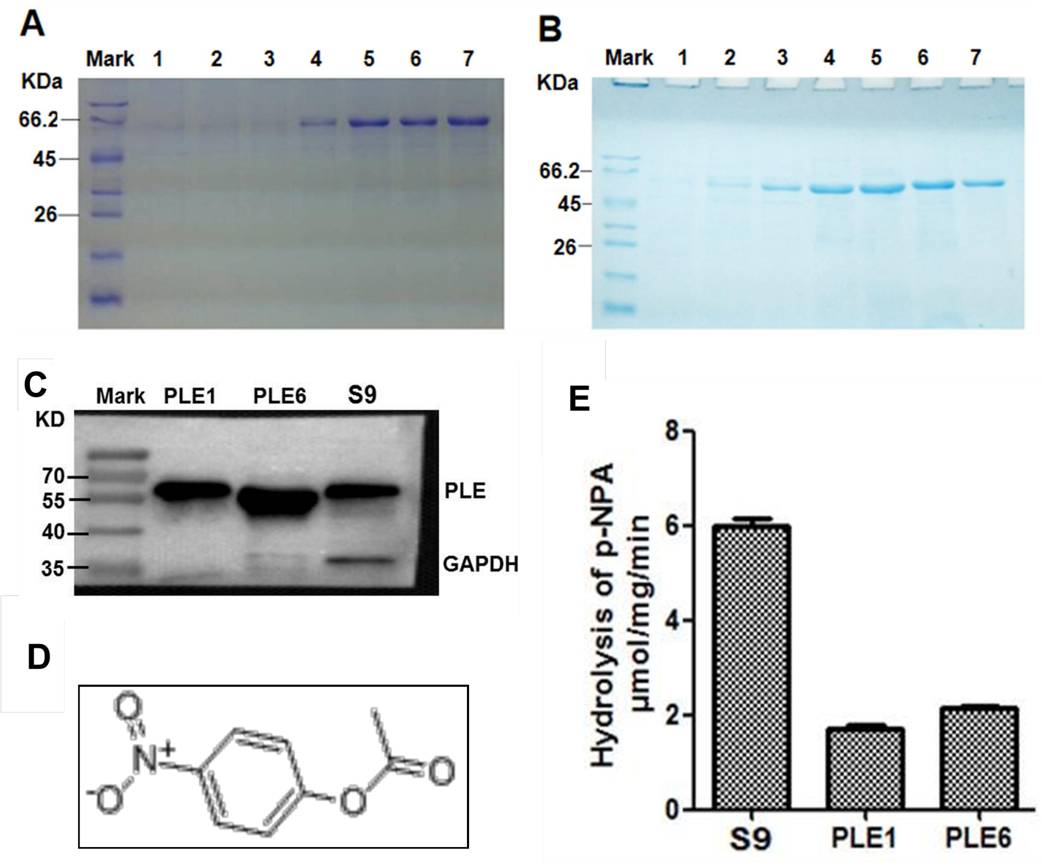

Fig. S1 Detection of the expression levels and hydrolytic activities of purified PLEs and pig liver S9 fractions. (A, B) The purity of His-tag-purified recombinant PLE1 and PLE6 was analyzed with SDS-PAGE. Lanes 1-7 represent the elution buffer with different concentrations. **(C)** Western blot detected the purified PLE1 (5 μg), PLE6 (10 μg) and liver S9 fractions (50 μg). **(D)** The structure of *p*-NPA. **(E)** Hydrolytic activities. Substrate *p*-NPA (200 μM) was prepared in 990 μl of reaction buffer Tris-HCl (50 mM, pH 7.4) and then mixed with purified PLEs (10 μg) or liver S9 fractions (100 μg). Hydrolytic activities were spectrophotometrically determined. Data in Fig. S1E are presented as the mean ± SEM of 3 independent experiments.

Supplemental Figure 2

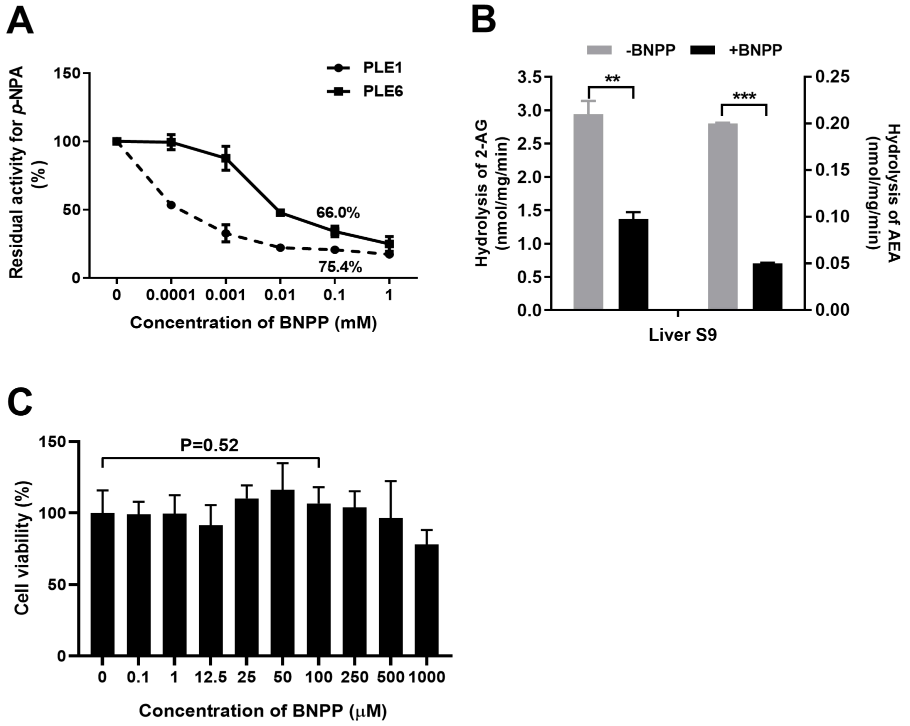

Fig. S2 Effect of BNPP on the hydrolytic activities of PLEs for *p*-NPA and cell viability. (A) BNPP inhibited the hydrolytic activities of recombinant PLE1 and PLE6 for *p*-NPA. **(B)** Hydrolysis activities of liver S9 for 2-AG and AEA. **(C)** The effect of BNPP (0.1-1000 μM) on cell viability. Cells (293T) were cultured in 96-well plates at a density of 10,000 cells/well. After an additional 12 h incubation, cells were treated with BNPP at various concentrations (0.1-1000 μM) for 36 h. Cell viability was determined with a CCK-8 assay as described in the "Supplemental Materials and Methods". The cell viability was expressed as the percentage of cells incubated without BNPP. The data in Fig. S2 are presented as the mean ± SEM of 3 independent experiments. Statistical significance was considered at values of $P < 0.05$ and indicated by an asterisk (* $P < 0.05$; ** $P < 0.01$; *** $P < 0.001$).

Supplemental Figure 3

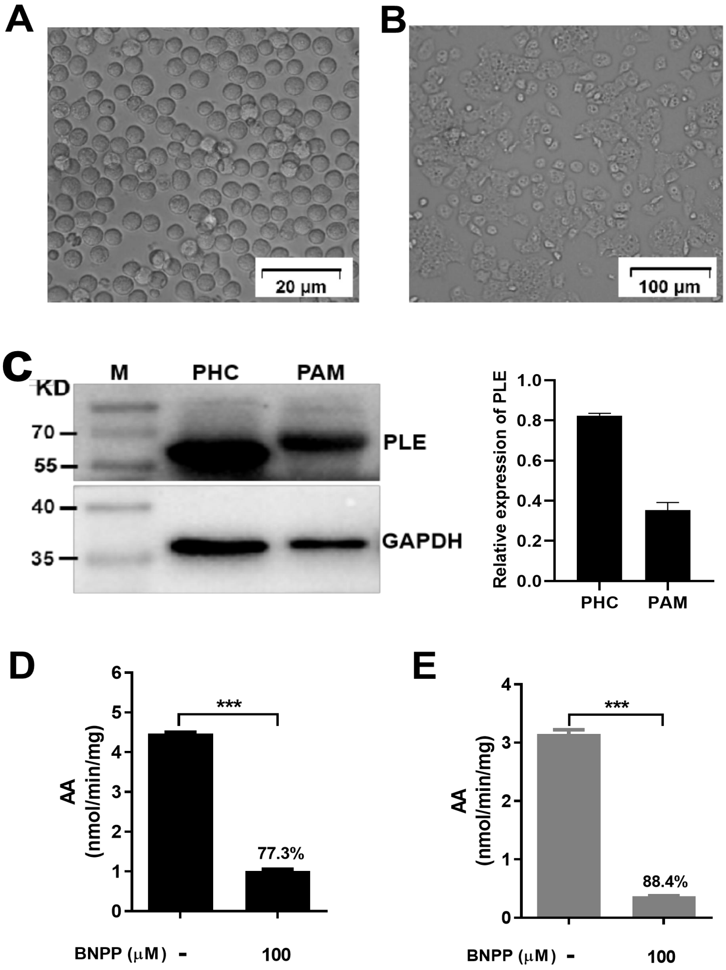

Fig. S3 Detection of the PLE level and hydrolytic activity for 2-AG in PAMs and PHCs. (A, B) Morphological analysis of PAMs (A) and PHCs (B) cultured for 12 h. **(C)** The expression levels of PLEs in PHCs and PAMs were detected by western blot. **(D, E)** The contribution percentage of PLEs for 2-AG hydrolysis in PAM (D) and PHC (E) homogenates, respectively. PAMs and PHCs were cultured for 24 h, and the cell lysates were collected. 2-AG (200 μM) was prepared in Tris-HCl buffer (50 mM, pH 7.4) in a total volume of 100 μL, and reactions were initiated by the addition of PAM or PHC homogenates (20 μg). Alternatively, PAM/PHC homogenates (20 μg) were added to Tris-HCl buffer along with BNPP (100 μM). After preincubation for 10

min at 37 °C, reactions were initiated by the addition of 2-AG (200 μM). After further incubation, all these reactions were terminated, and the free AA was detected with LC-MS/MS. Data in Fig. S3 D and E are presented as the mean ± SEM of 3 independent experiments. Statistical significance was considered at values of $P < 0.05$ and indicated by an asterisk (* $P < 0.05$; ** $P < 0.01$; *** $P < 0.001$).

Supplemental Figure 4

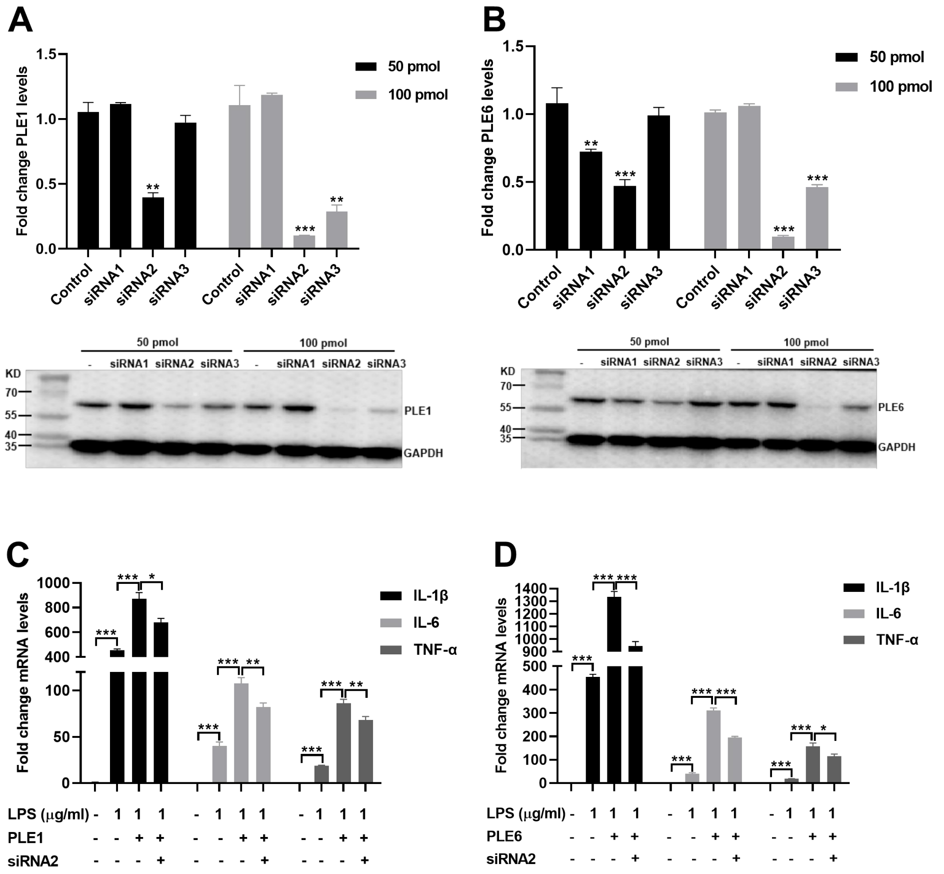

Figure S4 Detection the proinflammatory cytokines and PLEs in coculture model of PAM and PLE-transfected 293T cells. (A, B) Effect of corresponding siRNA on the the expression of PLE1 (A) and PLE6 (B) in PLE-transfected 293T cells. **(C, D)** Detection the proflammatory cytokines in coculture model of PAM and PLE-transfected 293T cells with RT-qPCR. PLE-transfected 293T cells were transfected siRNAs targeting PLEs for 24 h and then treated with 1 μg/ml LPS for 6 h. The total RNA or cells lysates were collected for RT-qPCR and western blot analysis, respectively. The data in Fig. S5 are presented as the mean ± SEM of 3 independent experiments. Statistical significance was considered at values of $P < 0.05$ and indicated by an asterisk (* $P < 0.05$; ** $P < 0.01$; *** $P < 0.001$).

Supplemental Figure 5

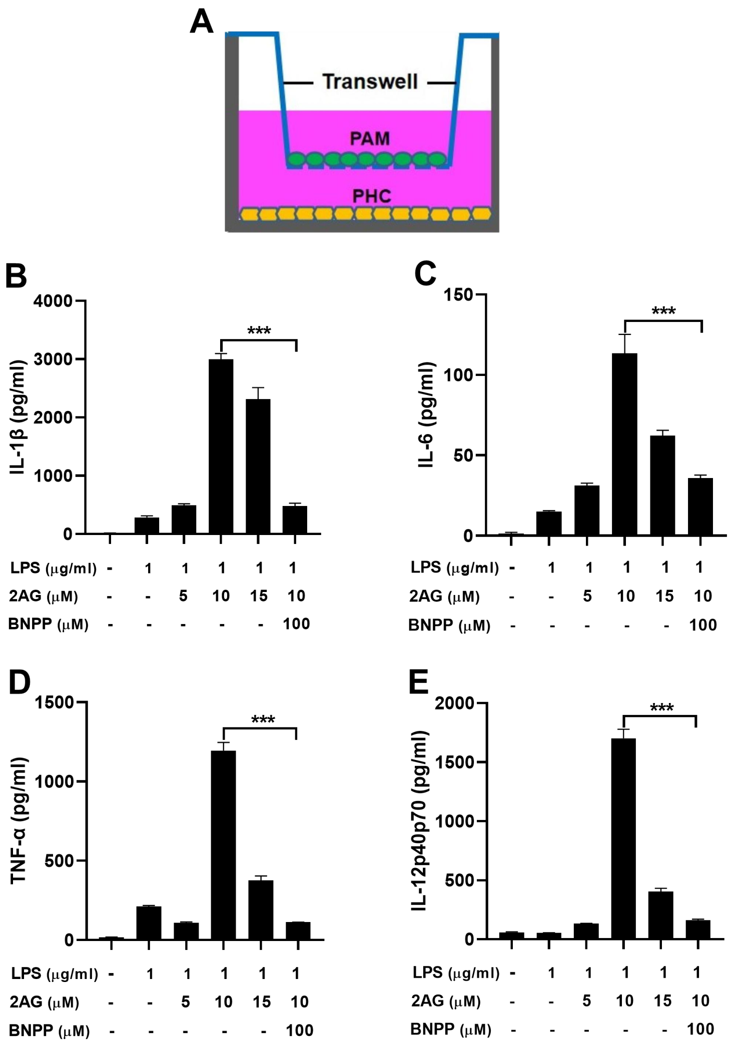

Figure S5 Detection of pro-inflammatory factors in co-culture model of PAM and PHC with protein chip. (A) Double-layered co-culture model diagram of PAM and PHC. **(B, C, D, E)** Detection of IL-1β (B), IL-6 (C), TNF-α (D) and IL-12p40p70 (E) in co-culture supernatants with protein chip. PAM and PHC were co-cultured for 24 h, including LPS, 2-AG, BNPP, and the culture supernatants were harvested and detected by protein chip. The data in Fig. S6 are presented as the mean ± SEM of 3 independent experiments. Statistical significance was considered at values of P < 0.05 and indicated by an asterisk (* P < 0.05; ** P < 0.01; *** P < 0.001).

Supplemental Figure 6

```
hCE1       MWLRAFILATLSASAAWAGHPSSPPVVDTVHGKVLGKFVSLEGFAQPVAI   50
PLE6       MWLLPLVLTSLASSATWAGQPASPPVVDTAQGRVLGKYVSLEGLAQPVAV   50
PLE1       MWLLPLVLTSLASSATWAGQPASPPVVDTAQGRVLGKYVSLEGLAQPVAV   50
Consensus  mwl    l  l  sa wag p sppvvdt  g vlgk vsleg aqpva

hCE1       FLGIPFAKPPLGPLRFTPPQPAEPWSFVKNATSYPPMCTQDPKAGQLLSE  100
PLE6       FLGVPFAKPPLGSLRFAPPQPAEPWSFVKNTTSYPPMCCQDQLGEQMLSD  100
PLE1       FLGVPFAKPPLGSLRFAPPQPAEPWSFVKNTTSYPPMCCQDPVVEQMTSD  100
Consensus  flg pfakpplg lrf ppqpaepwsfvkn tsyppmc qd    q  s

hCE1       LFTNRKENIPLKLSEDCLYLNIYTPADLTKKNRLPVMVWIHGGGLMVGAA  150
PLE6       LFTNRKERLIPEFSEDCLYLNIYTPADLTKRGRLPVMVWIHGGGLVVGGA  150
PLE1       LFTNGKERLTLEFSEDCLYLNIYTPADLTKRGRLPVMVWIHGGGLVLGGA  150
Consensus  lftn ke      sedclylniytpadltk  rlpvmvwihgggl  g a

hCE1       STYDGLALAAHENVVVVTIQYRLGIWGFFSTGDEHSRGNWGHLDQVAALR  200
PLE6       STYDGLALAAHENVVVVAIQYRLGIWGFFSTGDEHSRGNWGHLDQVAALH  200
PLE1       PMYDGVVLAAHENVVVVAIQYRLGIWGFFSTGDEHSRGNWGHLDQVAALH  200
Consensus    ydg  laahenvvvv iqyrlgiwgffstgdehsrgnwghldqvaal

hCE1       WVQDNIASFGGNPGSVTIFGESAGGESVSVLVLSPLAKNLFHRAISESGV  250
PLE6       WVQENIANFGGDPGSVTIFGESAGGESVSVLVLSPLAKNLFHRAISESGV  250
PLE1       WVQENIANFGGDPGSVTIFGESAGGESVSVLVLSPLAKNLFHRAISESGV  250
Consensus  wvq nia fgg pgsvtifgesaggesvsvlvlsplaknlfhraisesgv

hCE1       ALTSVLVKKGDVKPLAEQIAITAGCKTTTSAVMVHCLRQKTEEELLETTL  300
PLE6       AFTAGLVRK.DMKAAAKQIAVLAGCKTTTSAVFVHCLRQKSEDELLDLTL  299
PLE1       ALTVALVRK.DMKAAAKQIAVLAGCKTTTSAVFVHCLRQKSEDELLDLTL  299
Consensus  a t  lv k d k  a qia  agcktttsav vhclrqk e ell  tl

hCE1       KMKFLSLDLQGDPRESQPLLGTVIDGMLLLKTPEELQAERNFHTVPYMVG  350
PLE6       KMKFFALDLHGDPRESHPFLTTVVDGVLLPKMPEEILAEKDFNTVPYIVG  349
PLE1       KMKFLTLDFHGDQRESHPFLPTVVDGVLLPKMPEEILAEKDFNTVPYIVG  349
Consensus  kmkf  ld  gd res p l tv dg ll k pee  ae  f tvpy vg

hCE1       INKQEFGWLIPMQLMSYPLSEGQLDQKTAMSLLWKSYPLVCIAKELIPEA  400
PLE6       INKQEFGWLLPT.MMGFPLSEGKLDQKTATSLLWKSYPIANIPEELTPVA  398
PLE1       INKQEFGWLLPT.MMGFPLSEGKLDQKTATSLLWKSYPIANIPEELTPVA  398
Consensus  inkqefgwl p   m   plseg ldqkta sllwksyp   i  el p a

hCE1       TEKYLGGTDDTVKKKDLFLDLIADVMFGVPSVIVARNHRDAGAPTYMYEF  450
PLE6       TDKYLGGTDDPVKKKDLFLDLMGDVVFGVPSVTVARQHRDAGAPTYMYEF  448
PLE1       TDKYLGGTDDPVKKKDLFLDLMGDVVFGVPSVTVARQHRDAGAPTYMYEF  448
Consensus  t kylggtdd vkkkdlfldl  dv fgvpsv var hrdagaptymyef

hCE1       QYRPSFSSDMKPKTVIGDHGDELFSVFGAPFLKEGASEEEIRLSKMVMKF  500
PLE6       QYRPSFSSDKKPKTVIGDHGDEIFSVFGAPFLRGDAPEEEVSLSKMVMKF  498
PLE1       QYRPSFSSDKKPKTVIGDHGDEIFSVFGFPLLKGDAPEEEVSLSKTVMKF  498
Consensus  qyrpsfssd kpktvigdhgde fsvfg p l   a eee  lsk vmkf

hCE1       WANFARNGNPNGEGLPHWPEYNQKEGYLQIGANTQAAQKLKDKEVAFWTN  550
PLE6       WANFARSGNPNGEGLPHWPMYDQEEGYLQIGVNTQAAKRLKGEEVAFWND  548
PLE1       WANFARSGNPNGEGLPHWPMYDQEEGYLQIGVNTQAAKRLKGEEVAFWND  548
Consensus  wanfar gnpngeglphwp y q egylqig ntqaa  lk  evafw

hCE1       LFAKKAVEKPPQTEHIE                                   567
PLE6       LLSKEAAKKPPKIKHAE                                   565
PLE1       LLSKEAAKKPPKIKHAE                                   565
Consensus  l  k a  kpp   h e
```

Figure S6 The sequence alignment analysis between PLE6, hCE1 and PLE1 by DNAMAN. The protein accession numbers are, hCE1: NP_001020366, PLE6: AQT33900.1, PLE1: NP_999411.

Supplemental Table 1

Table S1 Molecular properties of hCE1, hCE2, PLE1 and PLE6 (4-7).

Property	hCE1	hCE2	PLE1	PLE6
Endoplasmic reticulum retention signal	HIEL	HTEL	HAEL	HAEL
Catalytic triad	Ser^{222}-Glu^{355}-His^{469}	Ser^{228}-Glu^{345}-His^{468}	Ser^{222}-His^{467}-Glu^{470}/Asp^{116}	Ser^{222}-His^{467}-Glu^{470}/Asp^{116}
Hydrolyzed characteristics	hCE1 prefers to metabolize the esters with a small alcohol group and a large bulky acyl group.	hCE2 prefers to hydrolyse esters with a relatively large alcohol group and a small acyl group.	PLE1 prefers to hydrolyse esters with a large alcohol group and a small acyl group.	PLE6 prefers to metabolize the esters that contain a small alcohol group and a large bulky acyl group.
Amino acid sequence identity	**PLE6 and hCE1: 77.9%;**<br>PLE6 and hCE2: 44.9%;<br>PLE1 and hCE1: 76.8%;<br>PLE1 and hCE2: 44.5%;<br>hCE1 and hCE2: 47.0%;<br>PLE1 and PLE6: 95.8%;			

Reference

1. Xiao, Q., Zhou, Q., Yang, L., Tian, Z., Wang, X., Xiao, Y., & Shi, D. (2018) Breed Differences in Pig Liver Esterase (PLE) between Tongcheng (Chinese Local Breed) and Large White Pigs. Scientific Reports **8**, 16364.
2. Böttcher, D., Brüsehaber, E., Doderer, K., and Bornscheuer, U. T. (2007) Functional expression of the gamma-isoenzyme of pig liver carboxyl esterase in Escherichia coli. Applied Microbiology & Biotechnology **73**, 1282-1289.
3. Shi, D., Yang, J., Yang, D., Lecluyse, E. L., Black, C., You, L., Akhlaghi, F., and Yan, B. (2006) Anti-influenza prodrug oseltamivir is activated by carboxylesterase human carboxylesterase 1, and the activation is inhibited by antiplatelet agent clopidogrel. Journal of Pharmacology & Experimental Therapeutics **319**, 1477-1484.
4. Imai T. Human carboxylesterase isozymes: catalytic properties and rational drug design. (2006) Drug Metab Pharmacokinet **21**:173–85.
5. Furihata T, Hosokawa M, Koyano N, Nakamura T, Satoh T, Chiba K. Identification of di-(2-ethylhexyl) phthalate-induced carboxylesterase 1 in C57BL/6 mouse liver microsomes: purification, cDNA cloning, and baculovirus-mediated expression. (2004) Drug Metab Dispos **32**:1170–7.
6. Bencharit S, Morton CL, Hyatt JL, Kuhn P, Danks MK, Potter PM, et al. Crystal structure of human carboxylesterase 1 complexed with the Alzheimer's drug tacrine: from binding promiscuity to selective inhibition. (2003) Chem Biol **10**:341–9.
7. Bencharit S, Morton CL, Howard-Williams EL, Danks MK, Potter PM, Redinbo MR. Structural insights into CPT-11 activation by mammalian carboxylesterases. (2002) Nat Struct Biol **9**:337–42.
